# Immunological Distinctions between Acellular and Whole-Cell Pertussis Immunizations of Baboons Persist for at Least One Year after Acellular Vaccine Boosting

**DOI:** 10.3390/vaccines8040729

**Published:** 2020-12-02

**Authors:** Leah E. Cole, Jinrong Zhang, Kristl M. Pacheco, Philippe Lhéritier, Natalie G. Anosova, Julie Piolat, Lingyi Zheng, Nathalie Reveneau

**Affiliations:** 1 Sanofi Pasteur, Cambridge, MA 02139, USA; jinrongzhang@hotmail.com (J.Z.); Kristl.Pacheco@sanofi.com (K.M.P.); Natalie.Anosova@sanofi.com (N.G.A.); 2 Sanofi Pasteur, 69280 Marcy L’Etoile, France; Philippe.Lheritier@sanofi.com (P.L.); Julie.Piolat@sanofi.com (J.P.); 3 Sanofi Pasteur, Swiftwater, PA 18370, USA; Lingyi.Zheng@sanofi.com

**Keywords:** pertussis, baboon model, immunological profile, whole cell pertussis vaccine, acellular pertussis vaccine

## Abstract

While both whole-cell (wP) and acellular pertussis (aP) vaccines have been highly effective at reducing the global pertussis disease burden, there are concerns that compared to wP vaccination, the immune responses to aP vaccination may wane more rapidly. To gain insights into the vaccine elicited immune responses, pre-adult baboons were immunized with either aP or wP vaccines, boosted with an aP vaccine, and observed over a nearly two-year period. Priming with a wP vaccine elicited a more Th17-biased response than priming with aP, whereas priming with an aP vaccine led to a more Th2-biased response than priming with wP. These differences were maintained after aP vaccine boost immunizations. Compared to aP, animals primed with a wP vaccine exhibited greater numbers of pertussis specific memory B cells. While aP and wP vaccine priming initially elicited similar levels of anti-pertussis toxin antibody, titers declined more rapidly in aP vaccine primed animals leading to a 4-fold difference. Both wP and aP vaccine immunization could induce serum bactericidal activity (SBA); however, only one wP vaccine immunization was required to elicit SBA while multiple aP vaccine immunizations were required to elicit lower, less durable SBA titers. In conclusion, when compared to aP vaccine, priming with wP vaccine elicits distinct cellular and humoral immune responses that persist after aP vaccine boosting.

## 1. Introduction

Pertussis (whooping cough) is an acute respiratory disease caused by the exclusively human Gram-negative bacterium *Bordetella pertussis*. Infants (<1 year old) are at greatest risk for severe disease manifestation and mortality due to bronchopneumonia [1]. Pertussis is a highly contagious disease that can affect all age groups and is increasingly being recognized as a cause of prolonged cough in both adolescents and adults in which the complications can include breakage of ribs and loss of consciousness due to violent coughing [2,3]. The introduction of whole-cell pertussis (wP) vaccines in the 1940s led to >99% decrease in the incidence of pertussis among young children. Though wP vaccines can be highly efficacious, concerns about injection-site reactions and systemic adverse events led to reduction in their use in several countries. This decline prompted the development and subsequent introduction of acellular pertussis (aP) vaccines that consist of purified components of the bacterium in the 1980s and 1990s. Clinical studies have shown that aP vaccines are safe, immunogenic, and efficacious [4,5]. Despite high rates of vaccination, many countries have experienced a steady increase in the reported cases of pertussis [6,7,8] since this switch. In 2012, the Centers for Disease Control and Prevention (CDC) reported 48,277 cases of pertussis in the United States, the highest in 50 years. During recent epidemics, increased rates of disease have been observed among adolescents that had been immunized solely with aP vaccines. While multiple factors, including enhanced disease awareness, increased case reporting due to more sensitive diagnostics, lack of booster immunizations, genetic changes in circulating strains, and the impact of past vaccination coverage could explain the recent resurgence of pertussis, it appears that the waning immunity and sub-optimal immune skewing may represent contributing factors [6,9,10,11,12,13,14,15,16].

Our goal was to examine immune signatures associated with the aP and wP vaccine priming and boosting over an extended period of time. While there is not an established serologic correlate of protection for pertussis, there is considerable clinical evidence supporting the critical role of antibodies in protection against disease [17,18] and preclinical studies have shown that antibodies alone are sufficient to protect against disease symptoms [19,20,21]. Other preclinical studies have indicated that T cells are involved in protection against bacterial colonization [22,23,24,25]. As humoral and cellular immune responses appear to be important for pertussis vaccine mediated protection, we determined that both would be assessed as part of this study. The baboon (*Papio Anubis*) preclinical model of pertussis is unique as it appears to recapitulate the full spectrum of human disease symptoms [26,27]. Therefore, baboons were selected to conduct this long-term investigation of vaccine elicited immune responses.

**Figure 1:**
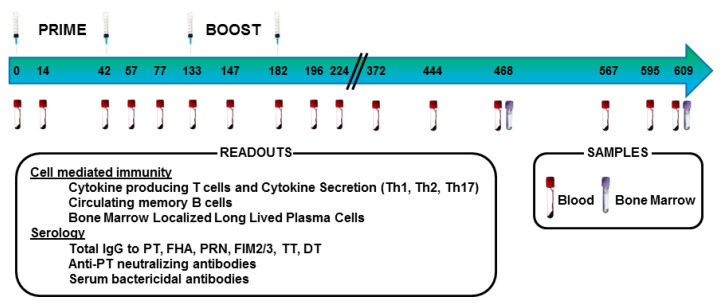
Immunization and sample collection timeline.

Immune responses were assessed in pre-adult baboons (aged 4–5 years at the initiation of the study) that received two primary immunizations with either an aP or wP vaccine followed by two boost immunizations with an aP vaccine (Figure 1). We observed these baboons for over a year after the final boost immunization. This study did not include a bacterial challenge, therefore direct conclusions about bacterial colonization and disease protection were not possible. Rather, this study was designed as an in-depth examination of the immune responses to aP and wP immunization. While these results agreed with the published data [22,28] in terms of the Th bias of the response after priming immunizations, this longitudinal study revealed additional novel differences in the humoral and cellular responses. Furthermore, the different immune responses observed in animals primed with either wP or aP vaccine persisted after aP vaccine boost immunizations suggesting the long-lasting impact of the primary immunization on the immune response to pertussis vaccination.

## 2. Materials and Methods

### 2.1. Ethics Statement

These studies did not include human subjects, therefore an ethical approval code was not needed; instead, all proposed animal procedures were reviewed and approved by the Texas Biomedical Research Institute (TBRI) and the Southwest National Primate Research Center (SNPRC) Institutional Animal Care and Use Committee (IACUC) for compliance with the Animal Welfare Act regulations (9 Code of Federal Regulations 1–4) prior to study initiation. This facility is accredited by Association for Assessment and Accreditation of Laboratory Animal Care (AAALAC) and licensed by the United States Department of Agriculture to conduct laboratory research using laboratory animals.

### 2.2. Animals, Immunization, and Sample Collection

Pre-adult Olive baboons and Olive baboon hybrids were 4–5 years old at the initiation of the study. All baboons tested negative for *Bordetella bronchiseptica* exposure before the start of the study. Six animals received 2 primary intramuscular immunizations with wP [DTwPfr (D.T.COQ/D.T.P, Sanofi-Pasteur Ltd., Marcy L’Etoile, France)] and six animals received aP [DTaP5cp (DAPTACEL, Sanofi-Pasteur Ltd., Toronto, ON, Canada)]. All animals were boosted twice with a modified Tdap vaccine containing 10 µg chemically detoxified pertussis toxoid (PT), 5 µg filamentous haemagglutinin (FHA), 5 µg pertactin (PRN), 7.5 µg fimbrae (Fim 2/3), 5 LF tetanus toxoid (TT), 2 LF diphtheria toxoid (DT) and 330 µg aluminum hydroxide. Primary immunizations took place on study days 0 and 42 and boost immunizations occurred on study days 133 and 182 (Figure 1).

### 2.3. Collection of Blood, PBMC, and Bone Marrow

To obtain sera, blood samples were collected from the femoral veins and placed into serum separator tubes (SST). The SST were then centrifuged at 1800× *g* for 15 min at room temperature. Processing always occurred within two hours of collection. Serum samples were collected and stored frozen until needed for use in assays. Blood for peripheral blood mononuclear cells (PBMCs) samples was collected from a peripheral femoral vessel using sodium heparin tubes. PBMCs were purified using Leucosep™ tubes according to the manufacturer’s protocol. PBMC samples were stored frozen in FBS + 10% DMSO until assessment in T or B cells ELISPOT assays. On the indicated day, 10 mL of bone marrow was collected from proximal humerus bones using heparinized syringes. After transfer into sodium heparin blood tubes, mononuclear cells were purified from bone marrow using Leucosep™ tubes according to the manufacturer’s protocol.

### 2.4. Cytokine Detection in ELISPOT Assays

Prior to performing any ELISPOT assays, a stock of frozen heat-killed *Bordetella pertussis* (HKBp) strain 10536 was generated. This was done to maintain reagent consistency between assays. To create this stock, bacteria were grown first on solid Bordet Gengou plates and then in modified Stainer and Scholte (SS-SAT) medium (Boston Bioproduct, Ashland, MA, USA, 2001SP) before being diluted and grown in modified SS-SAT medium a second time. This stock of bacteria was resuspended in DPBS and heated in a 65 °C water bath for 30 min to kill the bacteria. In order to assess the number of pertussis antigen-specific IFN-γ (Th1), IL-13 (Th2) and IL-17A (Th17) secreting cells, multiscreen 96-well filter PVDF membrane plates (EMD Millipore, Burlington, MA, USA, MSIPS4W10) were coated overnight with cytokine capture antibody: IFN-γ (Mabtech, Stockholm, Sweden, 3420-3), IL-13 (Mabtech, 3470-3) and IL-17A (Mabtech, 3520-3). PBMC were thawed in complete media [RPMI1640 (Gibco, Thermo Fisher Scientific-US, Waltham, MA, USA 22400105) +10% FCS (Cytiva, Marlborough, MA, USA, SH30073.03) + Penicillin-Streptomycin (Sigma-Aldrich, St. Louis, MO, USA, P4333)], assessed for viability, and stimulated with either 50 μg/mL of PT, FHA, Fim 2/3, PRN, or HKBp (50 CFU:1 PBMC ratio) at 37 °C, 5% CO_2_. ConA (Sigma-Aldrich, St. Louis, MO, USA, C5275) was used in all assays as a positive control to ensure cryopreserved PBMCs were able to respond to stimuli. Assessments were performed in duplicate. After 72 h of incubation, cells were lysed with sterile water and 1 μg/mL solutions of biotinylated detection antibodies were added: IFN-γ (Mabtech, 3420-6), IL-13 (Mabtech, 3470-6) and IL-17A (Mabtech, 3520-6). Streptavidin-AP (Southern Biotechnology, Birmingham, AL, USA, 7100-04) followed by BCIP/NBT substrate solution (Thermo Fisher Scientific-US, Waltham, MA, USA) was used to visualize the spots. Development was stopped by rinsing the wells with tap water. The plates were air-dried overnight in the dark at room temperature. A CTL ImmunoSpot Analyzer (Cellular Technology Limited, Shaker Heights, OH, USA) was used to scan, count, and QC all wells in each plate. The antigen specific counts per well were normalized by subtraction of the corresponding media control counts. Results are reported as the number of cytokine secreting cells per 10^6^ PBMC. While only HKBp stimulation data is shown, all data was incorporated into the principal component analysis (PCA). Alongside the T cell ELISPOT assays, cytokine secretion by PBMCs stimulated with either 50 μg/mL of PT, FHA, Fim 2/3, or PRN, or HKBp (50CFU:1 PBMCs ratio) or positive control 5 µg/mL ConA or negative control media alone was also assessed. In brief, 50 µL of stimuli containing media (RPMI1640 +10% FCS + Penicillin-Streptomycin) was combined with 50 µL of PBMC suspension at 4 × 10^6^/mL in a 96-well cell culture plate (Corning Inc., Corning, NY, USA, Costar 3595). The plates were placed in a 37 °C incubator with 5% CO_2_ and incubated for 72 h. Cell culture supernatants were collected and kept at ≤−70 °C until cytokine concentrations were assessed using the Milliplex MAP Kit Non-Human Primate Cytokine Magnetic Bead Panel Assay (Milliplex PRCYTOMAG-40K or PCYTMG-40k-PX23). Cytokine secretion data are not shown but were incorporated into the PCA.

### 2.5. Memory B Cell ELISPOT

PBMC were thawed in complete media (RPMI1640 +10% FCS + Penicillin-Streptomycin) was and assessed for viability. Cells were stimulated by incubation in complete media with 1 µg/mL R848 and 10 ng/mL IL-2 at 37 °C with 5% CO_2_ for 72 h. Forty-eight hours into this incubation, one day prior to the completion of the stimulation, multiscreen 96-well filter PVDF membrane plates (EMD Millipore, MSIPS4W10) were coated with a pertussis antigen pool (5 μg/mL of PT, FHA, Fim 2/3, and PRN) or 100 µL of 10 μg/mL Anti-human IgG mAb MT91/145 (Mabtech, 3850-3) in DBPS or 100 µL of DPBS as a negative control. After stimulation, PBMCs were washed twice to remove secreted antibodies, then activated cells were enumerated, and 200,000 (day 196) or 400,000 (days 468 and 609) PBMCs were added to each well coated with the pertussis antigen pool or DPBS and 20,000 (day 196) or 40,000 (days 468 and 609) cells were added to the anti-human IgG coated wells. All plates were incubated in a 37 °C incubator with 5% CO_2_ for 20 h. After incubation, the cell culture media was removed, and sterile water was added to lyse the cells. To visualize the spots, 1 µg/mL biotinylated anti-human IgG monoclonal antibody (mAb MT78/145, Mabtech, 3850-6), followed by Streptavidin-AP (Southern Biotechnology, 7100-04) and BCIP/NBT substrate solution (Thermo Scientific, 34042) were added. Development was stopped by rinsing the wells with tap water. The plates were air dried overnight in the dark at room temperature and a CTL ImmunoSpot Analyzer (Cellular Technology Limited) was used to scan, count and QC the wells. Results are shown as memory B cell per 10^6^ PBMC.

### 2.6. Bone Marrow Localized Long Lived Plasma Cells ELISPOT

Multiscreen 96-well filter PVDF membrane plates (EMD Millipore, MSIPS4W10) were coated with either pertussis antigen pool (5 μg/mL of PT, FHA, Fim 2/3, and PRN) or 10^9^ CFU/mL heat-killed *Bordetella pertussis* in 100 mL DPBS overnight. Cryopreserved mononuclear bone marrow cells were thawed, counted, and assessed for viability. Next, 4 × 10^5^ viable mononuclear bone marrow cells were added to each well and incubated in a 37 °C incubator with 5% CO_2_. Twenty hours post incubation, culture media was removed, and sterile water was added to each well to lyse the cells. 1 µg/mL biotinylated anti-human IgG mAb MT78/145 (Mabtech, 3850-6) was added followed by Streptavidin-AP (Southern Biotechnology, 7100-04) and BCIP/NBT substrate solution (Thermo Scientific, 34042) in order to visualize the spots. The development reaction was stopped by rinsing the wells with tap water. The plates were air dried overnight in the dark at room temperature and a CTL ImmunoSpot Analyzer (Cellular Technology Limited) was used to scan, count and QC the wells in each plate. Results for each baboon were expressed as the numbers of antibody secreting long-lived plasma cells per 10^6^ mononuclear bone marrow cells.

### 2.7. Serum IgG Quantification by Multiplex Antibody Assay for the Simultaneous Determination of Antibodies to Bordetella Pertussis Antigens, Diphtheria Toxoid and Tetanus Toxoid

Antigen-specific IgG antibodies were assessed using a multiplex MSD U-PLEX assay (Meso Scale Discovery, Rockville, MD, USA). Each antigen was biotinylated and linked to unique spots onto the U-plex plates via specific linkers. Uplex plates were coated with PT (5 μg/mL), PRN (5 μg/mL), FHA (25 μg/mL), Fim2/3 (3 μg/mL), Diphtheria Toxoid (DT) at 2 µg/mL and Tetanus Toxoid (TT) at 2 µg/mL. Serial diluted samples of serum were assessed. The amount of IgG bound to each antigen was determined using anti-IgG conjugated to an electrochemiluminescent label (sulfo-tag-conjugated anti-HU/NHP IgG antibody from MSD). The chemiluminescent signal was measured using an MSD instrument. A control baboon serum was generated from animals immunized with an aP vaccine. This control was used as a reference to calculate the titer for each sample.

### 2.8. Serum Bactericidal Activity (SBA) Assay

To assess SBA, baboon sera were heated for 30 min at 56 °C to inactivate complement. These samples were serially diluted two-fold (10 times) in microplates in Dulbecco’s phosphate buffered saline containing Ca++ and Mg++, in a volume of 50 µL. Next, 25 µL of a log phase *B. pertussis* bacterial suspension that had been grown for 48 h on solid Bordet Gengou (BG) + 20% sheep blood followed by 20 h in liquid SS-SAT medium and then adjusted to 10^4^ CFU/mL was added to each well. The SBA assay was performed using *B. pertussis* 18323 strain in the presence of 25% precolostral calf serum. After 60 min of incubation at +36 °C with shaking, 30 µL of the mixture from each well was plated onto BG + 20% sheep blood agar plate and incubated for 3 days at +36 °C in a 5% CO_2_ atmosphere. The colonies were counted, and the bactericidal titer was reported as the highest reciprocal serum dilution at which ≥50% killing of bacteria was observed as compared to the complement control. Controls included in each experiment consisted of assessment of bacterial viability when exposed to the complement source but not antibodies (complement control), bacteria and heat-inactivated complement (bacteria control), bacteria and heat-inactivated complement in the presence of antibodies (serum control).

### 2.9. Pertussis Toxin Neutralizing Antibody Titration

PT neutralizing antibody titers were assessed in a Chinese hamster ovary (CHO) cell assay, as previously described [29]. In brief, five microliters (5 µL) of diluted baboon sera (1/20) were added to 96-well microplates in the first well and then 2-fold serially diluted. Next 50 µL of PT (Biological Reference Preparation (BRP) Batch1.2 European Directorate for the Quality of Medecines (EDQM—Y0000021) at 2.5 ng/mL, equivalent to 125 pg of PT per well, was added. This amount is deleterious for the monolayer and induces formation of clusters throughout the entire well. Microplates were then incubated at +37 °C, 5% CO_2_ for 2 to 3 h. Following this incubation, 100 µL containing 5000 Chinese Hamster Ovary (CHO) K1 cells (American Type Culture Collection CCL‑61) were added per well and the microplates were incubated at +37 °C, 5% CO_2_ for 48 h. At the end of the incubation, cells were fixed with methanol and stained by addition of Giemsa solution. The reading of the microplates was performed visually under a microscope. The neutralizing titer was reported as the reciprocal serum dilution in the last well with a monolayer (no cluster observed). Samples with titers below the assay cut-off of 20 were arbitrarily attributed a titer of 10 (50% of the cut-off). Several controls were included in each test. There was a cell monolayer control with medium only to verify the quality of the monolayer. A PT control was included to verify the activity of the toxin. Both a neutralizing mAb 7E10 (NIBSC 99/542) and a pool of neutralizing mouse sera (against genetically detoxified PT) were included as positive controls. The negative control was a pool of non-neutralizing sera from naïve mice.

### 2.10. Statistical Analysis

The T memory cell response, including Th1/Th2/Th17, generated after a priming immunization with either aP or wP vaccine were compared using Analysis of Variance (ANOVA). When comparing the pertussis-specific circulating memory B cells ELISPOT data on study days 196, 468, and 609 between 6 aP-Prime/aP-Booster and 6 wP-Prime/aP-Booster animals, the number of days post immunization (a numerical variable) was potentially a factor. Therefore, the Analysis of Covariance (ANCOVA) model was applied using the vaccine group used to prime animals (aP vs. wP) as the fixed factor and study day as the covariate that accounted for the repeated measurements of each animal. The Bone Marrow pertussis-specific circulating memory B cells between aP-Prime/aP-Booster vs. wP-Prime/aP-Booster on single study day 609 was compared using Analysis of Variance (ANOVA).

When comparing the specific IgG antibody (to PT, FHA, PRN and Fim 2/3 antigens) of U-Plex (MesoScaleDiscovery, MSD) results, PT neutralization titer and SBA titers between 6 aP-Prime/aP-Booster and 6 wP-Prime/aP-Booster animals, Analysis of Variance (ANOVA) was used on individual study days from D14 to D196. Additionally, ANCOVA models was used only for data between D196 and D609 using vaccine group as fixed factor and study days as covariate that accounted for the repeated measurements of each animal.

A multivariate approach using Principal Component Analysis (PCA) was conducted using the study day 57, 196, or 609 data. The multivariate PCA effectively reveals the internal structure of the data as it provides a low-dimension plot (i.e., dimension of 2) by projecting the most informative view angle of the data. All available variables at each time point were used and could include B cell ELISPOT, T cell ELISPOT, cytokine secretion, IgG antibody titer, PT neutralization titer and SBA titer data. Table S1 lists the variables included in each PCA.

## 3. Results

### 3.1. Animals Primed with wP Vaccine Exhibited a More Th17 Biased Response than Animals Primed with aP Vaccine While Priming with aP Vaccine Led to a More Th2 Biased Response than wP Vaccine Priming

**Figure 2:**
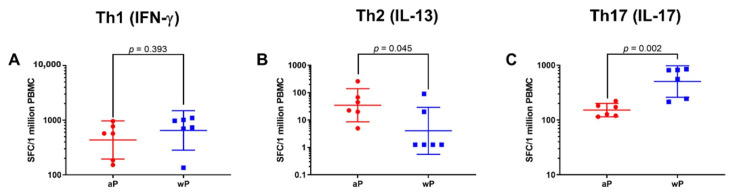
Assessment of the day 57 T cell response revealed differences in the Th bias in animals primed with wP (blue square) or aP (red circle) vaccines. The data shown in the IFN-γ (**A**), IL-13 (**B**) and IL-17 (**C**) graphs are the individual values from day 57 with the geometric means and 95% CI noted with the corresponding lines. Statistical analysis was performed using an ANOVA model with the specific vaccine used to prime animals (aP or wP) as the fixed factor. There was a statistically significant difference between wP vaccine and aP vaccine primed animals in the Th2 (IL-13) response (*p* = 0.045) and Th17 (IL-17) response (*p* = 0.002), but not for the Th1 (IFN-γ) response (*p* = 0.393).

To assess the T cell response and its helper profile elicited by priming with either a wP or aP vaccine, ELISPOT assays were performed using PBMC samples collected on day 57, approximately 2 weeks after the second primary immunization. The numbers of IFN-γ, IL-13, and IL-17 secreting cells were assessed as these cytokines are characteristic of Th1, Th2, and Th17 responses respectively. The data generated after recall with HKBp is displayed in Figure 2. Animals primed with a wP vaccine had significantly greater numbers of IL-17 producing cells compared to the aP vaccine primed group (wP geometric mean, 505.3 and 95% CI, 260.5–980.1 vs. aP geometric mean, 152.0 and 95% CI, 114.7–201.4). High numbers of IFN-γ producing cells were detected after recall with HKBp in samples from both aP and wP vaccine immunized animals; while the numbers were greater in the wP vaccine immunized baboons, the difference was not statistically significant (wP geometric mean of 650.4 and 95% CI, 283.6–1491.8 vs. aP geometric mean of 435.6 and 95% CI, 194.9–973.2). In contrast, priming with aP vaccine induced significantly greater numbers of IL-13 producing cells than immunization with wP vaccine (aP geometric mean 34.8 and 95% CI, 8.6–140.2 vs. wP geometric mean 4.05 and 95% CI, 0.6–29.1). Together, these data support the conclusion that priming with a wP vaccine elicits a more Th17 biased response than aP vaccine while priming with an aP vaccine provokes a more Th2 biased response than a wP vaccine.

### 3.2. T Cell Polarization Established by Vaccine Priming Is Maintained Post aP Vaccine Boost

**Figure 3:**
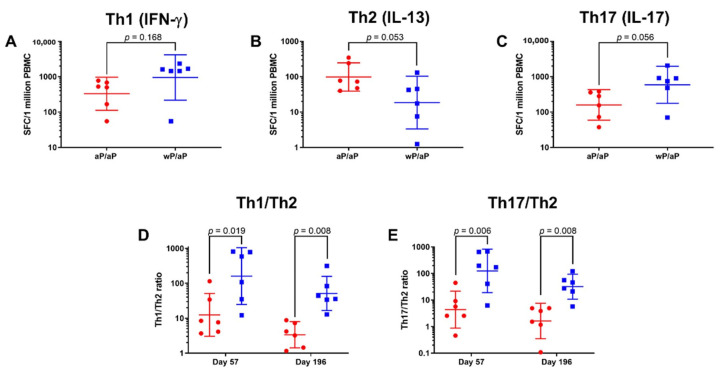
Assessment of T Cell Responses on Day 57 and Day 196 indicates durability of immune skewing after aP vaccine boosting. The data shown in the IFN-γ (**A**), IL-13 (**B**) and IL-17 (**C**) graphs are the individual values from day 196 with the geometric means and 95% CI noted with the corresponding lines. The Th1/Th2 and Th17/Th2 ratios were calculated based on the numbers of IFN-γ, IL-13, and IL-17 secreting cells after recall stimulation of PBMC with heat killed *B. pertussis* on days 57 and 196. Animals that were aP vaccine primed (day 57) or aP vaccine primed and aP vaccine boosted (day 196) are represented by red circles while animals that were wP vaccine primed (day 57) or wP vaccine primed and aP vaccine boosted (day 196) are represented by blue squares. The data shown in the graphs are the Th1/Th2 (**D**) or Th17/Th2 (**E**) ratios and the individual calculated values are shown with the geometric means and 95% CI noted with the corresponding lines.

To assess the impact of an aP vaccine boost on the recall of established memory T cells and their helper profile, PBMC samples were analyzed on day 196. Samples were stimulated with HKBp and the numbers of IFN-γ (Th1), IL-13 (Th2), and IL-17 (Th17) secreting cells were assessed (Figure 3A–C). The results on day 196 were similar to what was seen on day 57 despite all animals receiving an aP vaccine for the boost immunizations. There were greater number of IL-17 (wP/aP geometric mean, 591 and 95% CI, 177–1972 vs. aP/aP geometric mean, 159 and 95% CI, 59–430) and IFN-γ (wP/aP geometric mean, 950 and 95% CI, 217–4157 vs. aP/aP geometric mean, 329 and 95% CI, 112–971) secreting cells in the animals that were primed with wP vaccine and boosted with aP vaccine when compared to animals that were primed and boosted with aP vaccine. Greater numbers of IL-13 secreting cells were observed in animals primed and boosted with aP vaccine when compared to animals that were primed with wP vaccine and boosted with aP vaccine (wP/aP geometric mean, 19 and 95% CI, 3–103 vs. aP/aP boost geometric mean, 98 and 95% CI, 39–246). To visualize the impact of the aP vaccine boost immunizations, the numbers of cells from both days 57 (2 weeks post 2 primes) and 196 (2 weeks post 2 boosts) were used to calculate the Th1/Th2 and Th17/Th2 ratios (Figure 3D,E). The Th profiles observed 2 weeks post priming with either wP or aP vaccine were maintained after the aP vaccine boosts (Figure 3). The geometric mean of the Th1/Th2 ratio at day 57 for aP vaccine primed animals was 12.5 (95% CI, 3.1–51.4); in contrast the geometric mean for the wP vaccine primed animals was 160.7 (95% CI, 24.7–1045.5). This difference was maintained after aP vaccine boosting as the geometric means of the Th1/Th2 ratios on day 196 for aP primed and aP boosted animals was 3.4 (95% CI, 1.4–8.0) while it was 51.2 (95% CI, 16.6–157.6) for wP vaccine primed and aP vaccine boosted animals. For the Th17/Th2 ratio, the geometric means on day 57 were 124.9 (95% CI, 19.0–822.1) for the wP vaccine primed animals and 4.4 (95% CI, 0.9–21.6) for aP vaccine primed animals. On day 196 the geometric means for the Th17/Th2 ratios were 31.9 (95% CI, 10.7–94.7) for the wP vaccine primed and aP vaccine boosted animals while it was 1.6 (95% CI, 0.4–7.5) for aP vaccine primed and boosted animals. These Th1/Th2 and Th17/Th2 ratios indicate that the differential T-cell polarization established after priming is maintained after the aP vaccine boosts.

### 3.3. Priming with wP Vaccine Appeared to Increase the Size of Both the Pertussis Specific Circulating Memory B Cell and Bone Marrow Localized Long Lived Plasma Cell Populations Compared to aP Vaccine

**Figure 4:**
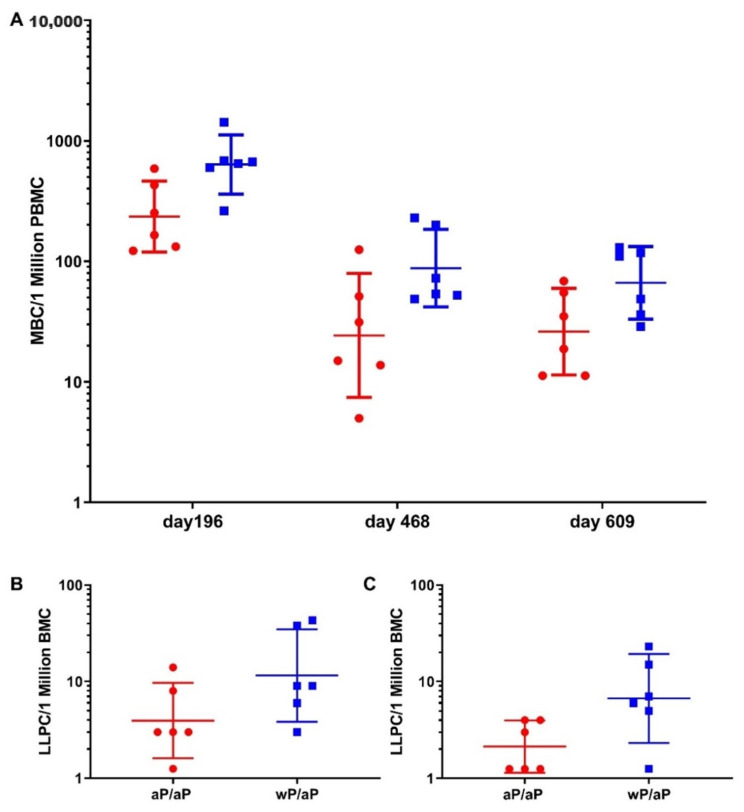
Compared with aP vaccine, priming with wP vaccine leads to an increased B cell response. The circulating memory B cell population (**A**) was assessed using ELISPOT plates coated with a pool of pertussis vaccine antigens (pertussis toxoid, FHA, PRN, and Fim2/3). Time of PBMC collection is indicated along the x-axis. ELISPOT plates coated with either a pool of pertussis vaccine antigens (**B**) or HKBp (**C**) were used to assess the antigen specific bone marrow localized long lived plasma cell population in samples collected on study day 609. Animals that were aP vaccine primed and aP vaccine boosted are represented by red circles while animals that were wP vaccine primed and aP vaccine boosted are represented by blue squares. The data points are values from individual animals with the geometric means and 95% CI noted with the corresponding lines. The memory B cell data were analyzed using an ANCOVA model with group as the fixed factor and time as the covariate. A repeated day option was used to consider the identical baboons by day/group. A global *p* value for the circulating memory B cells was calculated for days 196, 468, and 609 (*p* = 0.024). For the long-lived plasma populations, statistical analysis was performed using an ANOVA model with the aP vaccine primed and aP vaccine boosted and wP vaccine primed and aP vaccine boosted animals at day 609 as the fixed factor. While the difference between the pertussis vaccine antigen specific populations was not statistically significant (*p* = 0.094) the difference between the HKBp specific populations was statistically significant (*p* = 0.024).

To gain insight on the durability of cellular compartments associated with a long lasting humoral immune response, the pertussis-specific memory B cells and bone marrow localized long lived plasma cell populations were assessed after the final boost immunization. The pertussis-specific memory B cells were assayed using PBMCs that were collected on study days 196, 468, and 609 (Figure 4). There was a statistically significant difference in the responses in animals that were primed with aP vaccine and boosted with aP vaccine versus those that were primed with wP vaccine and boosted with aP vaccine at all three time points (*p* = 0.024). When the geometric means were compared, there were 2.67 (day 196), 3.52 (day 468), and 2.53 (day 609) fold more pertussis specific circulating memory B cells in wP vaccine primed and aP vaccine boosted animals in comparison to aP vaccine primed and aP vaccine boosted animals. Bone marrow localized long lived plasma cells were also assessed. On study day 609, priming with wP vaccine led to greater numbers of pertussis vaccine antigen specific long-lived plasma cells, the geometric means were 11.4 (95% CI, 3.6–35.6) for the wP vaccine primed and aP vaccine boosted animals and 3.6 (95% CI, 1.2–11.2) for the aP vaccine primed and aP vaccine boosted animals. This difference did not reach statistical significance (*p* = 0.094) (Figure 4). The number of long-lived plasma cells specific for whole bacteria was also assessed. Priming with wP vaccine led to greater numbers HKBp specific long-lived plasma cells when compared to the aP vaccine primed animals and this difference was statistically significant (*p* = 0.024).

### 3.4. Higher Anti-PT IgG Response Sustained in wP-Primed Baboons Compared to aP-Primed for 14 Months Following Boost

The magnitude and the durability of the total IgG antibodies specific to FHA, PT, PRN, Fim2/3, DT, and TT was assessed. Serum samples were collected before the first injection, as well as 14 days and approximately 40 days following each of the four immunizations and at multiple time points up to 14 months after the final immunization.

**Figure 5:**
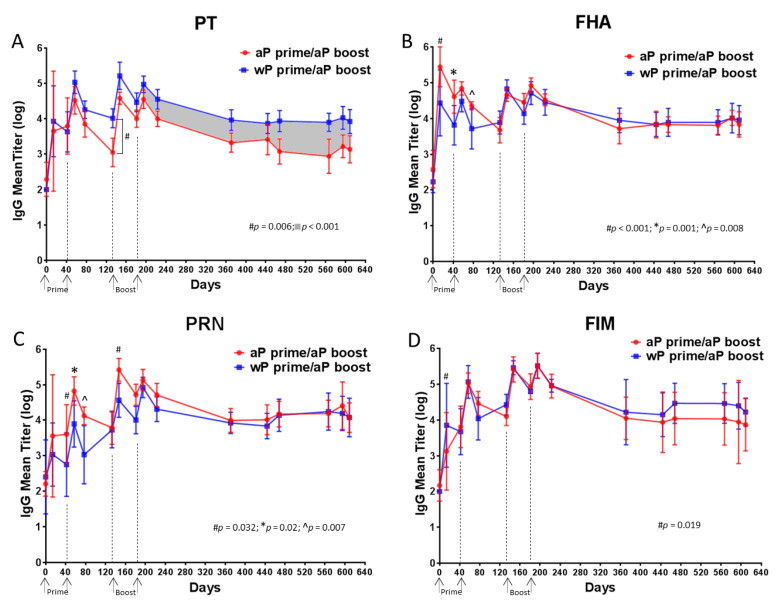
Analysis of PT- (**A**), FHA- (**B**), PRN- (**C**), Fim 2/3 (**D**)-specific IgG antibodies revealed differences in durability and magnitude of response. The data shown in the graphs at each time point are the means of 6 individual values with 95% CI noted with the corresponding lines. A statistical analysis was performed using two different models: (i) ANOVA model with the specific primary vaccine immunization (aP or wP) as the fixed factor for each time point D14, D42, D57, D77, D133, D147, D181, D196. (ii) ANCOVA model with group as the fixed factor and time as the covariate from D196 to D609. The *p* values are listed on the specific graphs.

Figure 5 shows the total IgG responses to PT (panel A), to FHA (panel B), to PRN (panel C) and to Fim2/3 (panel D). Priming with either a wP or aP vaccine induced an anti-PT IgG response in all baboons, the magnitude of the response increased to average titers close to 4 logs for both vaccines (Figure 5A). These titers increased by 1 log after a second primary immunization with either vaccine. When assessed three months after this second primary immunization (day 133), the anti-PT IgG titer dropped by 1.5 log in aP vaccine primed baboons. In contrast, there was a smaller drop in titers in wP vaccine primed animals and the difference in titer at day 133 for the two groups of animals was statistically significant (*p* = 0.006). The aP vaccine booster immunizations increased the anti-PT titers in all animals. However, following the last booster immunization, the anti-PT IgG response decreased more rapidly in aP vaccine primed baboons than in wP vaccine primed baboons leading to a 4-fold difference in titer. A significantly higher anti-PT IgG response (*p* < 0.001) was sustained in wP vaccine primed baboons compared to aP vaccine primed animals for 14 months following the final boost. Taken together this suggests that wP vaccine priming elicits a more durable antibody response to PT than aP vaccine priming.

The response to FHA was different than what was observed with the anti-PT response. A greater magnitude response to FHA was observed in aP vaccine primed baboons compared to wP vaccine primed animals at the earlier time points (Figure 5B). The differences between aP vaccine primed and wP vaccine primed baboons were significant at days 14 (*p* < 0.001), 42 (*p* = 0.001) and 77 (*p* = 0.008), however these distinctions did not persist after the two booster immunizations and the responses were not distinguishable over the 14-month period following the final boost.

Similarly, the response to PRN was greater in aP vaccine primed baboons compared to wP vaccine primed animals at days 14 (*p* = 0.032), 42 (*p* = 0.02), 77 (*p* = 0.007) and 147 (*p* = 0.032) but differences were no longer detectable after two booster immunizations as there were no statistically significant differences in titer during the 14-month period following the final boost (Figure 5C).

The responses to Fim2/3 were similar for all animals throughout the experiment, the one exception being a single time point after the first immunization (day 14) for which the anti-Fim2/3 IgG titer was significantly higher in wP vaccine primed baboons than in aP vaccine primed baboons (Figure 5D).

Two non-pertussis antigens, DT and TT, are also components of the wP and aP vaccines and antibody titers to these antigens were also assessed (data not shown). Similar results were observed for both anti-DT and -TT titers. After priming, there were higher titers in animals that received aP vaccine immunizations, however after aP vaccine boosting, higher titers were observed in the wP primed animals (data not shown). There were higher anti-TT and -DT titers on study days 195–609 for the wP vaccine primed and aP vaccine boosted animals in comparison to the aP vaccine primed and aP vaccine boosted animals (*p* = 0.029 DT and *p* = 0.029 TT).

### 3.5. Compared to aP Vaccine Primed Baboons, wP Vaccine Primed Baboons Had Higher PT-Neutralizing Titers That Were Sustained over 14 Months Following aP Vaccine Boost

A clinical study involving 192 children showed a correlation between antibody binding titers obtained by ELISA and the toxin neutralization activity [30], however this study also showed that an antibody titer obtained by one method could generally not be used to predict a titer for the other method with accuracy at the level of an individual serum sample. Therefore, due to the importance of anti-toxin activity and the relatively small number of animals in this non-human primate (NHP) study, toxin neutralizing activity was assessed in addition to assessing the anti-PT antibody binding titers (Figure 5A).

**Figure 6:**
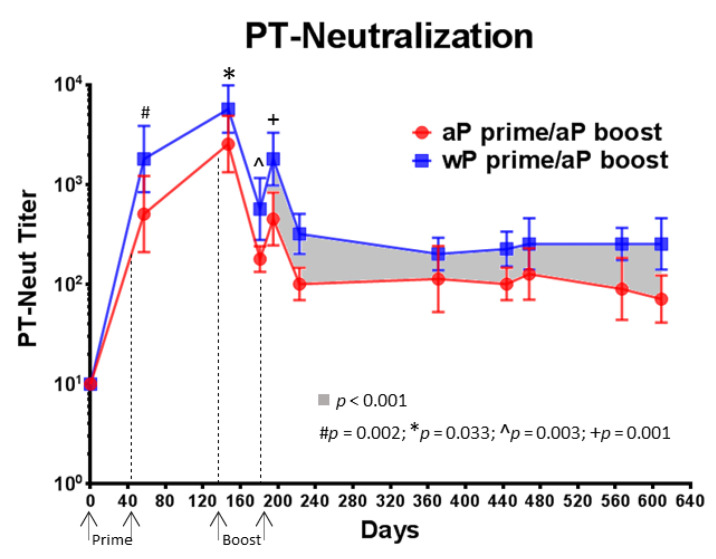
Priming with wP vaccine leads to elevated anti-PT neutralizing antibody titers. PT neutralization was assessed in the Chinese Hamster Ovary (CHO) cell cytotoxicity assay. The data shown at each time point are the mean values from 6 individual animals and the corresponding lines indicate the 95% CI. A statistical analysis was performed using two different models: (i) ANOVA model with the specific primary vaccine immunization (aP or wP) as the fixed factor for days 57, 147, 181, and 196. (ii) ANCOVA model with group as the fixed factor and time as the covariate for the interval between days 196 to 609. The *p* values are shown in the figure.

PT-neutralizing antibody activity was measured using individual serum samples collected before the first immunization as well as 14 days after each subsequent immunization. High PT-neutralization titers were obtained after the first immunization for both vaccines. Significantly higher titers at day 57 (*p* = 0.002), day 147 (*p* = 0.033), day 181 (*p* = 0.003) and day 196 (*p* = 0.001) were detected in wP vaccine primed baboons compared to aP vaccine primed baboons. Significantly higher PT-neutralizing titers (*p* < 0.001) were maintained over 14 months following the last boost immunization in wP vaccine primed and aP vaccine boosted animals when compared to aP vaccine primed and aP vaccine boosted baboons (Figure 6). This result indicates that a wP vaccine priming induces an anti-PT neutralizing response that is greater in magnitude and durability than the response elicited by aP vaccine priming. Individual PT-neutralizing titers and anti-PT IgG titers were compared on days 57, 195, and 609 and were found to correlate (data not shown).

### 3.6. A Single Immunization with wP Vaccine Is Sufficient to Induce a Serum Bactericidal Antibody Response While Multiple Immunizations with aP Vaccine Are Necessary

**Figure 7:**
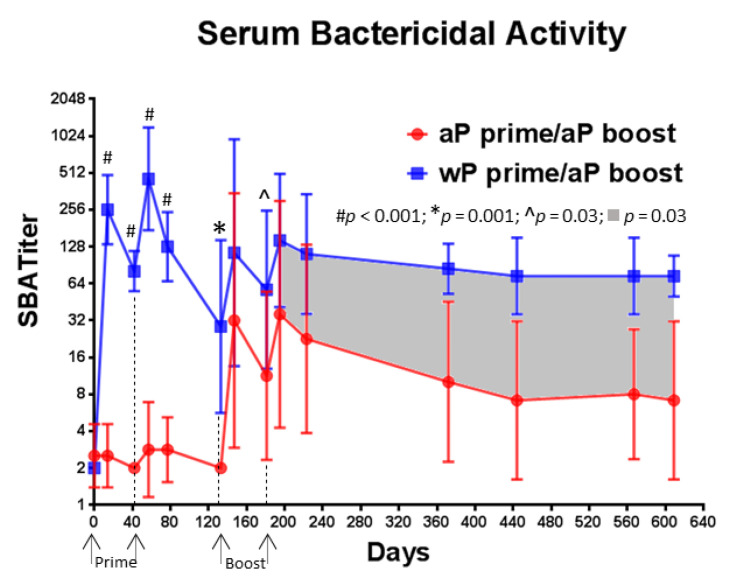
Priming with wP vaccine leads to higher serum bactericidal antibody titers. The serum bactericidal antibody response was measured in a classical killing assay against *Bordetella pertussis*. The data shown at each time point are the mean values from 6 individual animals and the 95% CI is noted with the corresponding lines. A statistical analysis was performed using two different models: (i) ANOVA model with the specific primary vaccine immunization (aP or wP) as the fixed factor for days 14, 42, 57, 77, 133, 147, 181, and 196. (ii) ANCOVA model with group as the fixed factor and time as the covariate for the interval between days 196 to 609. The *p* values are listed on the graph.

We evaluated the SBA of baboon serum samples collected throughout the study (Figure 7). High bactericidal activity was observed after the first immunization with wP vaccine in all baboons (mean titer = 256), while minimal, if any, activity was detected after the first immunization with the aP vaccine (mean titer = 2.5). The difference between the response induced by the two vaccines was highly significant on day 57 (*p* < 0.001). In contrast to what was observed with wP vaccine, multiple immunizations with aP vaccine were necessary to observe any increase in bactericidal activity and the titers were lower than what was observed in wP vaccine primed baboons (day 181 *p* = 0.03). When compared to aP vaccine primed baboons, the bactericidal antibody titer was consistently higher in wP vaccine primed animals for the 14 months following final aP vaccine boost immunization (*p* = 0.023). In addition, the SBA response declined more rapidly after the final boost immunization in aP vaccine primed animals than in wP vaccine primed baboons leading to a 6-fold difference in titer. These results suggest that when compared to an aP vaccine, a wP vaccine induces a rapid and durable functional antibody response that can kill *B. pertussis* in a complement dependent manner.

### 3.7. Principal Component Analysis (PCA) Reveals Immunological Distinction between Animals Primed with aP Vaccine Versus wP Vaccine

**Figure 8:**
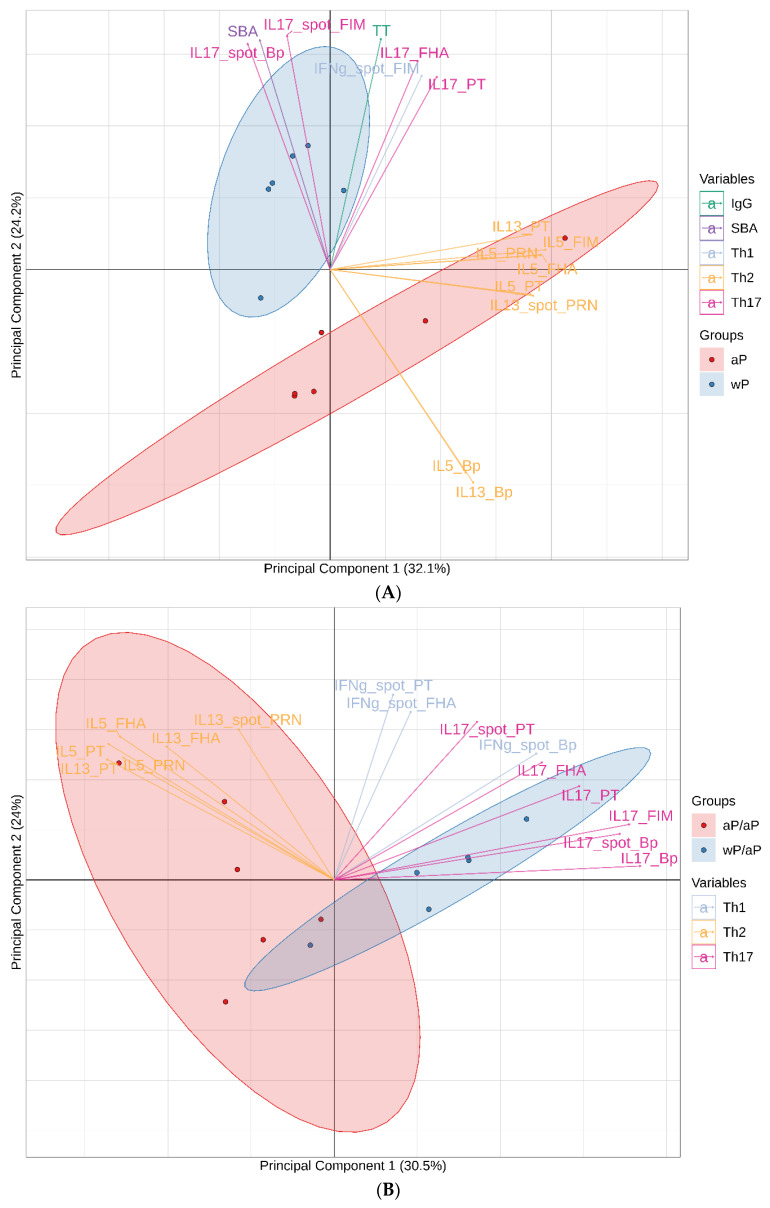

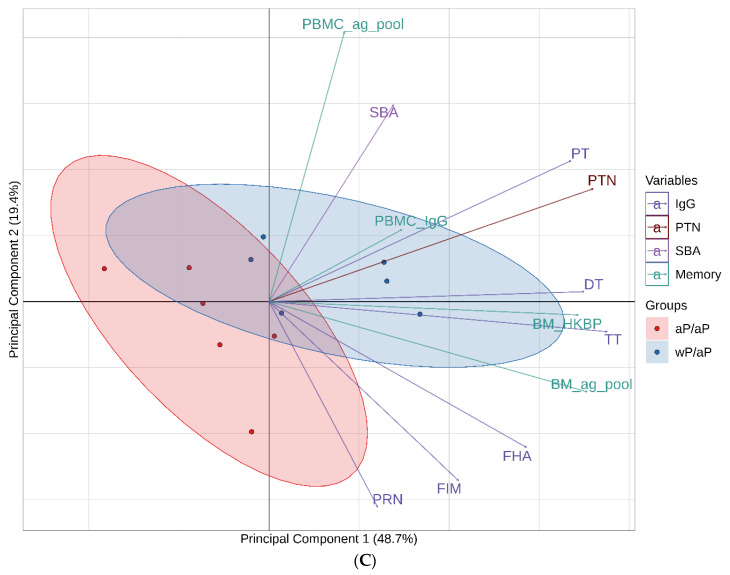
PCA of immune responses on days 57 (**A**), 196 (**B**), and 609 (**C**) reveals distinct immunological signatures. PCA was performed using data generated from the post primary immunizations day 57 samples as well as the post boost day 196 or 609 samples. There were 43 different variables incorporated into the day 57 PCA, 45 variables incorporated into the day 196 PCA, and 12 variables incorporated into the day 609 PCA. All variables included in the analyses are listed in Table S1. The most contributive variables to principal components 1 and 2 are listed in the biplots. For T cell ELISPOT data, variables are listed by the cytokine assessed, spot, and the specific recall antigen (e.g., IL17_spot_Bp). For T cell cytokine secretion after antigen recall the data is noted by the specific cytokine assessed followed by the recall antigen (e.g., IL17_Bp). For IgG antibody titers, data are noted solely by the name of the antigen (e.g., FHA). Serum bactericidal activity data is noted by SBA. PT-neutralizing activity data is noted by PTN (PT-neutralization). For B cell ELISPOT data, the source of the cells is listed as either bone marrow (BM) or PBMC along with the antigen specificity of the antibody produced by the cells.

To gain insight into the global immune response after immunization, multivariate PCA were performed. PCA is an effective way to reveal the internal structure of data as it can provide a low-dimension plot (i.e., dimension of 2) by projecting the most informative view angle of the data. These analyses incorporated data generated using the day 57 post primary immunizations samples and the day 196 and day 609 post boost immunizations samples (variable listed in Table S1). Principal components 1 and 2 are displayed for the day 57 (Figure 8A), day 196 (Figure 8B) and day 609 (Figure 8C) analyses. For the day 57 analysis, principal components 1 and 2 explain 56.3% of the total variation of the data. For the post boost analyses, principal components 1 and 2 explain 53.5% (day 196) and 68.2% (day 609) of the total variation of the data.

The day 57 PCA separated the wP vaccine and aP vaccine primed animals into 2 distinct groups (Figure 8A). The wP vaccine primed animals are clustered primarily in the second quadrant indicating a strongly correlated response to Th17 and SBA as well as a lack of skewing toward the Th2 response as the different Th2 variables were strongly negative contributors to component 2 as well as positive contributors to component 1. The aP vaccine primed animals cluster was spread through the first, third and fourth quadrants indicating that this group is defined by its Th2 response.

For the day 196 PCA, separation of the groups was primarily associated with the T cell responses. The aP vaccine primed and aP vaccine boosted subjects clustered in the second and third quadrants indicating a skew towards Th2 bias as Th2 variables are strong negative contributors to principal component 1. The wP vaccine primed and aP vaccine boosted animals clustered primarily in the first quadrant and indicating a strongly correlated response to Th17/Th1 variables as Th17/Th1 variables are strong positive contributors to principal components 1 and 2.

While T cell data were key contributors to the separation of aP vaccine and wP vaccine primed animals for the PCA performed with data from days 57 and 196, T cells were not assessed on day 609 (Table S1). On day 609, the variables incorporated into the 1st and 2nd principal components were antibody (titer and functionality), memory B cells, and long-lived plasma cells. Even without T cells in the analysis, the wP vaccine primed/aP vaccine boosted and aP vaccine primed/aP vaccine boosted groups were separated in PCA biplot based on magnitude differences in these cellular and antibody variables.

## 4. Discussion

The baboon model of pertussis infection is arguably the only available preclinical model that can recapitulate the characteristic human attributes of pertussis including paroxysmal coughing, mucus production, leukocytosis, and airborne transmission of infection [26,27]. Accordingly, this model is considered a unique and extremely important tool for the preclinical evaluation of pertussis vaccines and therapeutics. In this study, the humoral and cellular immune responses of pre-adult baboons that received 2 primary intramuscular immunizations with either a wP or aP vaccine and 2 boost immunizations with an aP vaccine were examined for over a year following the final immunization. This study provided an opportunity to examine a nearly two years long time course of immune responses elicited by different pertussis immunization regimens.

Our examination of the T cell responses on study day 57 (Figure 2) revealed that 2 primary immunizations with wP vaccine induced a more Th17 biased response than aP vaccine immunization and primary immunizations with aP vaccine led to a more Th2 biased response than wP vaccine immunization. Priming with wP vaccine elicited a greater number of T cells that produced the Th1 cytokine IFN-γ than aP priming, but the difference was not statistically significant. While these experiments involved pre-adult baboons and PBMC, the data agree with the results from prior infant baboon studies involving separated CD4+ T cells or transcriptomic analysis of whole blood [22,28]. Our study does not include a bacterial challenge, however in previous baboon studies it was shown that while convalescent, aP vaccine immunized, or wP vaccine immunized baboons are equally protected against disease; convalescent animals are not colonized in the nasopharynx upon rechallenge, wP vaccine immunization accelerates the clearance of bacteria after challenge, and aP vaccine immunization provides no advantage in the clearance of bacteria from the upper respiratory tract [22]. Taken together, these data provide support for the hypothesis that a Th17 response may be involved in the prevention or rapid clearance of *B. pertussis* infection of upper respiratory tract.

Baboons are not the only preclinical models that have shown a disparate impact of different vaccines on the immune response as well as challenge clearance. While wP vaccine immunization controlled bacterial shedding and could prevent transmission of *B. bronchiseptica* challenge in mice, aP vaccine only controlled disease and did not impact bacterial shedding or transmission [31]. Mice immunized with wP vaccine are protected against lung and nasal colonization, while aP vaccine immunization only protected against lung infection and failed to control infection in the murine nasal cavity [23,32]. The murine lung protective immunity induced by wP vaccine immunization is mediated largely by IFN-γ, while aP vaccine protective immunity in the lungs is dependent upon IL-17A [25]. Data suggest that IL-17-secreting respiratory tissue-resident memory CD4+ T cells might critical for protection against nasal colonization and wP vaccine immunized mice expand the population of IL-17 and IFN-γ-secreting T resident memory cells in lungs and nasal tissues after *B. pertussis* challenge, while aP vaccine immunization failed to promote either the accumulation or expansion of these cells [23]. Studies of the Th1 and Th2 cellular immune responses in infants and children have also shown an immune dichotomy as prior infection or whole cell vaccination are associated with Th1 responses while aP vaccines are associated with either Th2 or mixed Th1/Th2 responses [33,34,35,36,37].

The boosting of wP vaccine and aP vaccine primed animals with an aP vaccine enabled the examination of the immune imprinting concept in baboons. Human studies have shown that differential Th polarization resulting from primary immunization with either wP or aP vaccines persists even after boosting with an aP vaccine [38,39,40]. When immune responses were assessed in adults after an aP vaccine boost immunization, the CD4+ T cell response was Th2 skewed in the cohort that had been aP vaccine immunized as infants and Th1/Th17 skewed in the wP vaccine primed group [39]. The results from this study are consistent with these prior observations as the Th polarization established by vaccine priming was maintained after the aP vaccine boost immunizations (Figure 3). The similarity of the immune responses in both humans and baboons provides further support for the translational value of the baboon model for studying pertussis.

The pertussis antigen specific memory B cell population was assessed at multiple time points following the final boost immunization (Figure 4). There were consistently greater numbers of memory B cells in animals that received a wP vaccine as a primary immunization than in animals that received aP vaccine. This difference was also seen the in the long-lived plasma cells populations (Figure 4). These data suggest that wP vaccine priming may be more effective than aP vaccine priming at generating pertussis specific circulating memory B cell and bone marrow localized long lived plasma cells. These data are in alignment with the idea that wP vaccine immunization may provide more durable immunity. The increased numbers of long-lived plasma cells that recognize whole bacteria also suggest that wP vaccine immunization may elicit an immune response against a wider breadth of antigens than an aP vaccine.

Assessment of the serum antibody responses to pertussis vaccine antigens (Figure 5) revealed that while there were initially higher titers to FHA and PRN after aP vaccine priming, these distinctions were lost by the time of the first boost immunization. The antibody responses to Fim2/3 were similar for all animals throughout the experiment. In total, there was minimal if any distinction in the anti -FHA, -PRN, and -Fim2/3 titers for most of the time points examined. In contrast, while initially similar, the anti-PT IgG titers were lower after aP vaccine boosting and waned more rapidly in aP vaccine primed animals when compared to the wP vaccine primed baboons (Figure 5). These differences in pertussis specific antibody responses are similar to what has been found in an analysis of randomized clinical trials involving adolescents that had been primed with either wP or aP vaccines and then boosted with one of two licensed aP vaccines. The anti-PT responses in adolescents that were primed with aP vaccine were markedly lower (as much as 71%) than those that were primed with wP vaccine. In contrast, there was little if any distinction in the anti-FHA responses and anti-PRN responses were only minimally reduced in aP vaccine primed adolescents in some studies [41].

Pertussis is primarily a toxin mediated disease therefore in addition to anti-toxin antibody binding titers, toxin neutralization activity was also assessed. Priming with wP vaccine led to greater PT neutralizing activity than aP vaccine and these differences were maintained after aP vaccine boosting (Figure 6). These differences in anti-PT binding antibody titers and pertussis toxin neutralizing activity may be important as there are multiple lines of evidence that support the critical role of anti-PT antibodies in protection against pertussis disease. Monocomponent pertussis toxoid vaccines have been shown to be effective [42] and clinical studies have shown a correlation between titers of anti-PT antibodies and protection against pertussis [17,43]. Preclinical studies have also demonstrated the important role of anti-PT antibodies as maternal immunization of baboons with a monocomponent pertussis toxoid vaccine protects infant baboons against the development of symptoms despite having no impact on bacterial colonization [21]. Other baboon studies have shown that passive delivery of anti-pertussis toxin antibody can protect animals against disease symptoms [44]. Accordingly, induction of higher, more long-lasting titers against PT may increase the duration of protection against disease. While the primary immunization may have a long term impact on anti-PT titers, recent clinical studies indicate that use of a booster aP vaccine that contains genetically detoxified PT as opposed to chemically inactivated PT can elicit significantly higher and more durable anti-PT titers, increased anti-PT neutralizing activity, and significantly enhanced numbers of PT-specific IgG memory B cells. This impact has been shown in studies involving adults [45] and adolescents [46] indicating a potential benefit for the inclusion of genetically detoxified PT in booster vaccines for both wP and aP vaccine primed individuals.

A recent clinical case report suggested the potential importance of complement mediated bacterial killing in the control of pertussis disease [47]. Therefore, in addition to examining pertussis toxin neutralizing activity, the bactericidal activity of the serum was assessed. To the best of our knowledge, this is the first examination of *B. pertussis* SBA associated with the preclinical baboon model. While one primary immunization with wP vaccine was sufficient to induce serum bactericidal antibodies, SBA was detectable only after multiple immunizations with aP vaccines (Figure 7). The mean SBA titers induced by wP vaccine priming were greater than those induced by aP vaccine priming at all time points assessed. While the specific targets of these bactericidal antibodies were not investigated as part of this study, lipo-oligosaccharide (LOS) [48] has been proposed as a main target of bactericidal antibodies for *B. pertussis*. As LOS is present on the surface of *B. pertussis*, it is present in the wP vaccine formulation but is not a component of the aP vaccine. Other studies have indicated that antibodies to PRN, a component of both wP and some aP vaccines, are bactericidal [49]. There were high total IgG anti-PRN titers (Figure 5) after the primary aP vaccine immunizations, but SBA was only detected after aP vaccine boosting, suggesting that if anti-PRN antibodies are directly mediating the complement mediated killing, multiple immunizations are required to drive the response to the appropriate IgG subclass or to elicit antibodies to the PRN-specific bactericidal epitopes in sufficient amounts or with sufficient affinity to produce detectable activity. In prior studies, baboons directly immunized with PRN containing aP vaccines were not protected against bacterial colonization in the upper respiratory tract [20,22] and maternal immunization studies with PRN containing aP vaccine formulations showed no impact on colonization in infant baboons [20]. Additional studies could be executed to examine the relationship between antibody mediated bactericidal killing and colonization as components that strongly elicit this functionality could be incorporated into novel acellular pertussis formulations.

Unsurprisingly, this long-term kinetic immune analysis yielded a large volume of data. In order to meaningfully examine the immunological impact of wP vaccine versus aP vaccine immunization, three PCA were performed using data generated with the day 57 post primary immunization samples, the day 196 post boost samples, and the day 609 post boost samples (Figure 8). While univariate analyses revealed differences in some of these individual variables (e.g., Th skewing and SBA), multivariate PCA provided a holistic approach that revealed correlations between multiple variables to immunization group and allowed for dimensionality reduction by providing a low-dimension plot (i.e., dimension of 2). Hence the principal components resulting from these analyses summarized the information and identified the immunological signatures associated with the different immunization regimens. These PCA visually illustrated that primary immunization with either wP or aP imprints distinct immune attributes (Figure 8A) and these immune differences were maintained over time and even after aP vaccine boost immunizations (Figures 8B and 8C).

## 5. Conclusions

In conclusion, when compared to aP vaccine, primary immunization of baboons with wP vaccine leads to distinct humoral and cellular immune responses that persist even after aP vaccine boosting. In addition to impacting the cellular immune response in sustained manner, wP vaccine priming elicits functional antibodies more rapidly and durably than aP vaccine immunization. While both aP and wP vaccines have been shown to protect baboons against disease symptoms, the immunological differences observed in this study may provide potential new areas of immune functionality to target in the development of novel aP vaccine formulations.

## Supplementary Materials

The following are available online at https://www.mdpi.com/2076-393X/8/4/729/s1, Table S1. Variables for the PCA.
