# Supplementary material for: Immunological Distinctions between Acellular and Whole-Cell Pertussis Immunizations of Baboons Persist for at Least One Year after Acellular Vaccine Boosting
Source: Vaccines (Basel). 2020 Dec 2;8(4):729. doi: 10.3390/vaccines8040729 (PMC7761625; doi:10.3390/vaccines8040729)
Supplement: Supplementary file 1 [file vaccines-08-00729-s001.pdf]

Table S1. Variables for the PCA. A total of 43 variables per animal were incorporated into to the post prime, day 57 PCA. There were 45 different variables per animal incorporated into post boost analyses for the day 196 PCA. There were 12 different variables included in the day 609 PCA.

				**Included in PCA**		
**Assay**	**Type of Readout**	**Antigen Specificity/Recall**	**Number of readouts per time point per animal**	**Day 57**	**Day 196**	**Day 609**
**Pertussis specific Ab titers**	Total IgG	FHA, Fim2/3, PRN, PT, DT, and TT	6	X	X	X
**SBA**	SBA titer	N/A	1	X	X	X
**Pertussis Toxin Neutralization**	Neutralizing Titer	N/A	1	X	X	X
**Pertussis Specific Memory B cells**	Ab secreting cells/10(6) PBMC	Antigen pool (FHA, Fim2/3, PRN, and PT) and total IgG	2		X	X
**Pertussis Specific Long-Lived Plasma Cells**	Ab secreting cells/10(6) BM	Antigen pool (FHA, Fim2/3, PRN, and PT) and HKBp	2			X
**T cell ELISPOT**	Cytokine (IFN-γ, IL-13, IL-17) secreting cells/10(6) PBMC	HKBp, FHA, Fim2/3, PRN, and PT	15	X	X	
**T cell cytokine secretion**	Cytokine (IFN-γ, IL-5, IL-13, IL-17) concentration (pg/mL)	HKBp, FHA, Fim2/3, PRN, and PT	20	X	X	
